# Mix Infections of *Helicobacter pylori*: A Major Risk Factor Affecting Genotyping Studies

**DOI:** 10.1155/2014/302192

**Published:** 2014-06-05

**Authors:** Amin Talebi Bezmin Abadi

**Affiliations:** ^1^Department of Medical Microbiology, University Medical Center Utrecht, Heidelberglaan 100, 3584 CX Utrecht, The Netherlands; ^2^Department of Bacteriology, School of Medical Sciences, Tarbiat Modares University, P.O. Box 14115-111, Tehran, Iran

We read with interest the paper “*Evaluation of the pattern of EPIYA motifs in the Helicobacter pylori cagA gene of patients with gastritis and gastric adenocarcinoma from the Brazilian Amazon region*” in the volume 2014, 2014, issue of International Journal of Bacteriology [1]. In this paper, Vilar e Silva et al. [1] presented that EPIYA-C motif in the* cagA* gene was found linked with the development of intestinal metaplasia and gastric adenocarcinoma. Remarkably, the authors mentioned that they only used monoinfected subjects in this survey. As result, in this study, genomic DNA was extracted from the antral biopsy specimens using commercial kit, not from bacterial culture. Indeed, more than 5–10% of* H. pylori* cases are mix infections [2, 3]. Undeniably, having mix infections in* H. pylori* colonized persons is a kind of pitfall for all kinds of genotyping studies. That would be more reliable if authors extract DNA from bacterial culture rather than from biopsy specimen; subsequently, it is not exaggeration to say that the mix infections were almost ignored in this investigation. Interestingly, in this examination, authors found a significant association between EPIYA-C and occurrence of gastric adenocarcinoma among individuals with mix infections; ironically, we need to prove that those patients are mixed by several infections. Brazil as a country with high prevalence of* H. pylori* and* H. pylori-*induced diseases would be a novel area to investigate these critical virulence factors. Finally, we recommend the investigation of* H. pylori* EPIYA motifs among the strains isolated as single colony that surely result in trustful and reliable findings in the Brazilian population.
